# A High Affinity hRpn2-Derived Peptide That Displaces Human Rpn13 from Proteasome in 293T Cells

**DOI:** 10.1371/journal.pone.0140518

**Published:** 2015-10-14

**Authors:** Xiuxiu Lu, Fen Liu, Sarah E. Durham, Sergey G. Tarasov, Kylie J. Walters

**Affiliations:** 1 Protein Processing Section, Structural Biophysics Laboratory, Center for Cancer Research, National Cancer Institute, Frederick, Maryland, United States of America; 2 Middletown High School, Middletown, Maryland, United States of America; 3 Biophysics Resource, Structural Biophysics Laboratory, Center for Cancer Research, National Cancer Institute, Frederick, Maryland, United States of America; University of Minnesota, UNITED STATES

## Abstract

Rpn13 is a proteasome ubiquitin receptor that has emerged as a therapeutic target for human cancers. Its ubiquitin-binding activity is confined to an N-terminal Pru (pleckstrin-like receptor for ubiquitin) domain that also docks it into the proteasome, while its C-terminal DEUBAD (DEUBiquitinase ADaptor) domain recruits deubiquitinating enzyme Uch37 to the proteasome. Bis-benzylidine piperidone derivatives that were found to bind covalently to Rpn13 C88 caused the accumulation of polyubiquitinated proteins as well as ER stress-related apoptosis in various cancer cell lines, including bortezomib-resistant multiple myeloma lines. We find that a 38-amino acid peptide derived from the C-terminus of proteasome PC repeat protein hRpn2/PSMD1 binds to hRpn13 Pru domain with 12 nM affinity. By using NMR, we identify the hRpn13-interacting amino acids in this hRpn2 fragment, some of which are conserved among eukaryotes. Importantly, we find the hRpn2-derived peptide to immunoprecipitate endogenous Rpn13 from 293T cells, and to displace it from the proteasome. These findings indicate that this region of hRpn2 is the primary binding site for hRpn13 in the proteasome. Moreover, the hRpn2-derived peptide was no longer able to interact with endogenous hRpn13 when a strictly conserved phenylalanine (F948 in humans) was replaced with arginine or a stop codon, or when Y950 and I951 were substituted with aspartic acid. Finally, over-expression of the hRpn2-derived peptide leads to an increased presence of ubiquitinated proteins in 293T cells. We propose that this hRpn2-derived peptide could be used to develop peptide-based strategies that specifically target hRpn13 function in the proteasome.

## Introduction

The ubiquitin-proteasome pathway regulates protein degradation in eukaryotes, enabling orderly cell cycle progression, clearance of misfolded proteins, numerous signaling mechanisms, and general protein homeostasis. The 26S proteasome contains a catalytic core particle (CP) that is targeted by inhibitors approved for hematological cancers, including bortezomib and carfilzomib, as reviewed in [1].

Capped at either end of the CP is a 19S regulatory particle (RP) that houses ubiquitin receptors and processing enzymes, as well as a hexameric ring of ATPases. Ubiquitin chains are recognized in the proteasome by Rpn10/S5a [2] and Rpn13/Adrm1 [3, 4]. Human Rpn10 has two helical ubiquitin interacting motifs (UIMs) that adapt to bind ubiquitin chains [5, 6], whereas hRpn13 has been proposed to prefer K48-linked ubiquitin chains, based on the structure of its complex with monoubiquitin [4]. hRpn10 docks into the RP by using a separate VWA domain situated at its N-terminal end. Rpn13 binds ubiquitin chains with loops from an N-terminal Pru domain [3, 4], which also interacts with 100 kDa PC repeat protein proteasome component Rpn2/PSMD1 [7–9].

Rpn13 interaction with Rpn2 is well tailored to its function as a proteasome ubiquitin receptor. Different surfaces are used by the Pru domain for simultaneous binding of ubiquitinated substrates and proteasome [4], and interaction with Rpn2 activates Rpn13 for ubiquitin binding [10]. Rpn13 has a C-terminal DEUBAD (DEUBiquitinase ADaptor) domain [11] that binds to deubiquitinating enzyme Uch37 [7, 9, 12], one of three deubiquitinating enzymes in the RP. The Rpn13 DEUBAD domain also interacts intramolecularly with the Pru domain [10]. This interdomain interaction reduces Rpn13 affinity for ubiquitin [10]. Binding to hRpn2 abrogates the Pru:DEUBAD interaction, thus activating Rpn13 for ubiquitin [10].

Deletion of Rpn13 from mice is neonatal lethal [13], and loss of the Rpn10 UIMs leads to embryonic lethality [14], demonstrating that these two proteasome ubiquitin receptors cannot compensate for each other during development. The combined loss of Rpn13 and Rpn10 from murine liver results in accumulation of ubiquitin conjugates and also loss of ubiquitin shuttling factors at the proteasome [13]. These shuttling factors have N-terminal UBL domains that bind to the ubiquitin-binding domains of Rpn13 [3] and Rpn10 [15–17].

hRpn13 functions in ovarian and colorectal cancer proliferation, and its knockdown triggers apoptosis in these and other cancer cell lines [18–22]. RA190, a bis-benzylidine piperidone derivative that covalently attaches to Rpn13 Cys88, inhibits ubiquitin-mediated protein degradation and restricts growth of multiple myeloma and ovarian cancer xenografts [22]. Like hRpn2, RA190 abrogates Rpn13 interdomain interactions and this effect may contribute to its anti-cancer activity [22].

An *in vitro* GST pull-down assay demonstrated direct interaction between hRpn13 and hRpn2, with the hRpn13 Pru domain required and sufficient for this interaction and the C-terminus of hRpn2 required [7]. This binding site was narrowed down to the extreme C-terminal 20 amino acids in *Saccharomyces cerevisiae* Rpn2 [23]. Here, in an effort to pinpoint the hRpn13-binding site on hRpn2, we identify a peptide at the very C-terminus of hRpn2 that binds hRpn13 with a dissociation constant of 12 nM. We use NMR spectroscopy and cell biology to identify the amino acids in hRpn2 that are critical for hRpn13 assembly into the proteasome. Moreover, we find that the hRpn2-derived peptide can immunoprecipitate endogenous hRpn13 and displace it from proteasome.

## Materials and Methods

### Plasmids and antibodies

The DNA fragment encoding amino acid 916 to 953 of hRpn2 was amplified by PCR and cloned into the bacterial expression vector pRSET and mammalian expression vector p3XFLAG-CMV7.1. Antibodies used in this study include anti-FLAG (Sigma), anti-hRpn2 (cell signaling), anti-hRpt3 (Abcam), and anti-hRpn13 (Abcam).

### Immunoprecipitation and immunoblotting

293T cells (purchased from ATCC) were cultured in DMEM supplemented with 10% fetal bovine serum at 37°C in a humidified atmosphere and 5% CO_2_. Plasmid DNA was transfected into 293T cells using Lipofectamine2000 (Invitrogen) according to the manufacturer’s instruction. Cells were harvested by gentle centrifugation at 500g for 5 minutes at 4°C after washing with PBS, and cell pellets were resuspended in 1% Triton-TBS buffer (50 mM Tris, pH7.5, 150 mM NaCl, 1 mM EDTA and protease inhibitor cocktail) followed by rocking at 4°C for 30 minutes. The supernatant was collected following centrifugation at 16,000g for 10 minutes and incubated with antibodies at 4°C overnight. Protein G sepharose beads (Sigma) were used to pull down interacting proteins by 3-hour incubation at 4°C. After extensive washing with 1% Triton TBS buffer, SDS-PAGE loading buffer was used to elute proteins from beads for immunoblotting.

### Sample preparation

hRpn2 (916–953) was expressed in *Escherichia coli* as a fusion protein with GST and a PreScission protease cleavage site. Cells were lysed by sonication and cellular debris removed by centrifugation at 31,000g for 30 minutes. The cell lysate was incubated with glutathione S-sepharose resin and washed extensively with Buffer 1 (20 mM sodium phosphate, 300 mM NaCl, 2 mM DTT, pH 6.5). hRpn2 (916–953) was eluted from the resin and separated from GST by overnight incubation with 50 u/mL PreScission protease in Buffer 2 (20 mM sodium phosphate, 50 mM NaCl, 2 mM DTT, pH 6.5). Further purification was achieved by size exclusion chromatography with a Superdex 75 column on an FPLC system. ^15^N ammonium chloride, ^13^C glucose, and D_2_O were used for isotope labeling. hRpn13 (1–150) was prepared as described by [4] with a method similar to that described above for hRpn2, but with a histidine tag in place of GST and Talon resin used for affinity purification. PreScission protease was used for elution and removal of the tag and all buffers were identical. All NMR experiments were performed in Buffer 2, but with addition of 10% D_2_O and 0.1% sodium azide. Protein concentrations were estimated by using calculated extinction coefficients for each protein and absorbance at λ = 280nm. The hRpn2 (916–953):hRpn13 Pru domain complex was passed over a size exclusion column after mixing to remove any excess component. The molar ratio was then evaluated by LC-MS analysis and found to be 1:1 stoichiometry.

### Isothermal titration calorimetry (ITC) experiments

ITC was performed at 25°C on a MicroCal iTC200 system. hRpn13 (1–150) and hRpn2 (916–953) samples were dialyzed extensively against ITC buffer (20 mM sodium phosphate, 50 mM NaCl and 10mM βME [pH 6.5]). One aliquot of 0.5 μL followed by eighteen aliquots of 2.1 μL 200 μM hRpn2 (916–953) were injected at 1000 rpm into the calorimeter cell (volume 200.7 μL), which contained 20 μM hRpn13. Blank experiments were performed by replacing protein samples with buffer and this blank data was subtracted from the experiment data during analyses. The integrated interaction heat values were normalized as a function of protein concentration, and the data were fit with MicroCal Origin 7.0 software. Binding was assumed to be at one site to yield the binding affinity K_a_ (1/K_d_), stoichiometry and other thermodynamic parameters.

### NMR experiments

All NMR experiments were conducted at 25°C on Bruker Avance 600 or 700 MHz spectrometers equipped with cryogenically cooled probes. ^1^H, ^15^N, ^13^C HNCACO, HNCO, HNCACB, CBCACONH and 3D-dispersed NOESY (200 ms mixing) spectra were acquired on 0.6 mM ^15^N-, ^13^C-, and 70% ^2^H-labeled hRpn2 (916–953) or on a mixture of 0.7 mM ^15^N-, ^13^C-, and 70% ^2^H-labeled hRpn2 (916–953) and equimolar unlabeled hRpn13 Pru. ^1^H, ^13^C HSQC and 3D ^13^C-edited NOESY spectra were recorded on a mixture of 0.5 mM ^13^C-labeled hRpn2 (916–953) and equimolar unlabeled hRpn13 Pru with a NOESY mixing time of 150 ms or on 0.5 mM ^13^C-labeled hRpn2 (916–953) with a NOESY mixing time of 300 ms.

Chemical shift perturbation (CSP) analysis was done by comparing ^1^H, ^15^N HSQC experiments recorded on ^15^N labeled hRpn2 (916–953) alone and with equimolar unlabeled hRpn13 Pru. CSP values were calculated according to Eq 1, as described in [24].

$$CSP=\sqrt{0.2{\left(\Delta \delta_{N}\right)}^{2}+{\left(\Delta \delta_{H}\right)}^{2}}$$ (1)

Δδ_H_, change in proton value (in parts per million); Δδ_N_, change in nitrogen value (in parts per million).

^1^H, ^15^N heteronuclear NOE experiments were performed with a four second saturation transfer or control period at 600 (for hRpn2 (916–953)) or 700 (for hRpn2 (916–953):hRpn13 Pru domain) MHz on spectrometers equipped with cryogenically cooled probes. Sample concentrations of 0.2 mM (hRpn2 (916–953)) or 0.4 mM (hRpn2 (916–953):hRpn13 Pru) were used and hRpn2 (916–953) was ^13^C, ^15^N, and 70% ^2^H labeled; hRpn13 Pru was unlabeled. The hRpn2 (916–953):hRpn13 Pru sample was passed over a size exclusion column after mixing and LC-MS analysis determined the complex to be at 1:1 stoichiometry. The program relax [25] was used for data analysis.

## Results

### A 38-amino acid hRpn2-derived peptide that binds to the hRpn13 Pru domain

**Fig 1:**
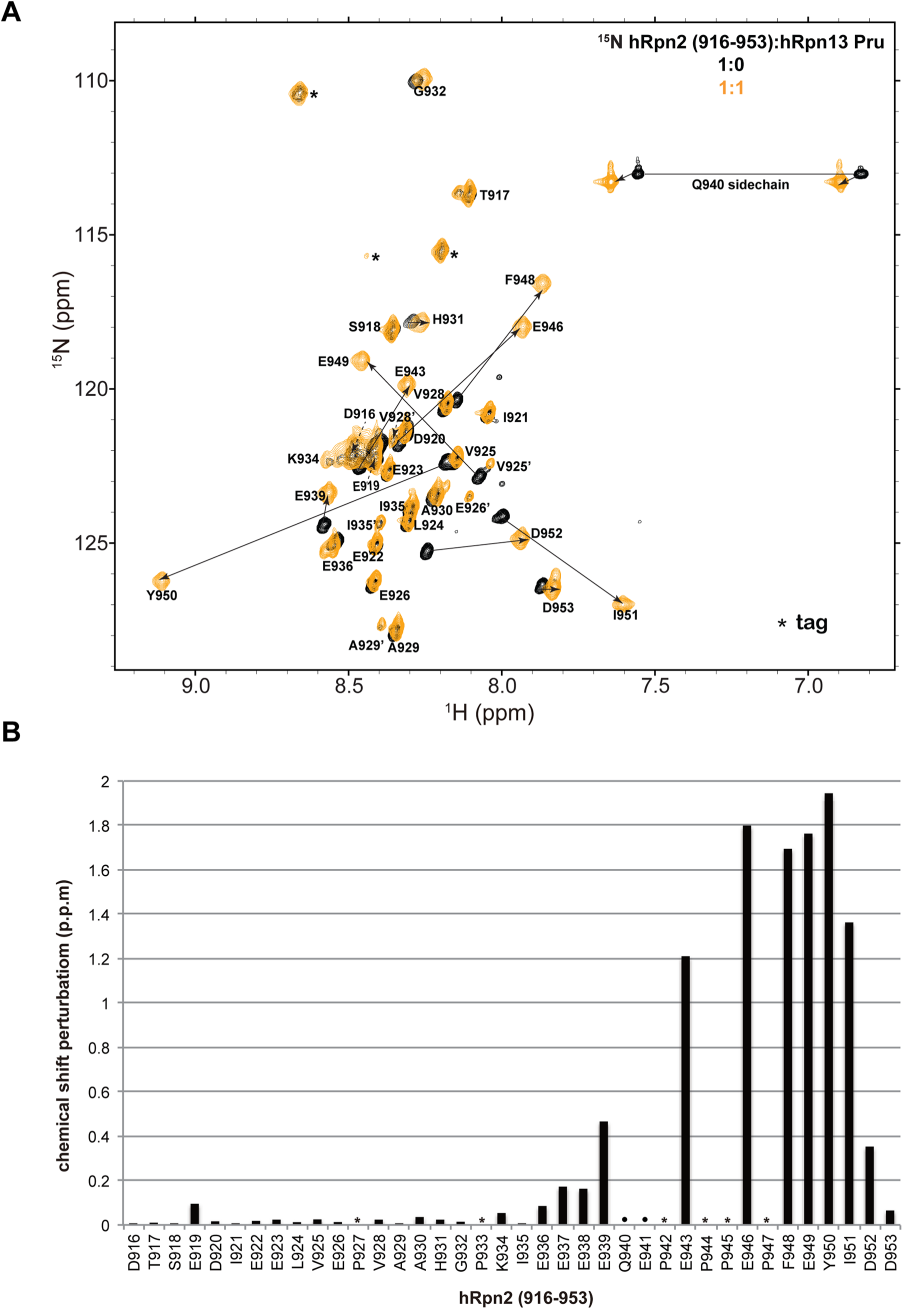
The C-terminal region of hRpn2 binds to the hRpn13 Pru domain. (A) ^1^H−^15^N HSQC spectra of hRpn2 (916–953) alone (black) and with equimolar Rpn13 Pru domain (orange). Amino acids from the tag are indicated with an asterisk. Signal shifting is indicated by a solid arrow that extends from the free state to the hRpn13-bound state. A dashed arrow is used for assignments in the congested central region of the spectra. Exchange signals are labeled with their amino acid identity followed by a prime signature (‘). All amino acids from the peptide were assigned except Q940 and E941; prolines lack amide protons and are excluded from this analysis. (B) Chemical shift perturbation (CSP) values derived from (A) for each hRpn2 amino acid, as described in Materials and Methods. Prolines, which lack amide signals, and unassigned Q940 and E941 are omitted from this analysis and indicated with an asterisk or circle, respectively.

In a previous study, we added a hRpn2 peptide that spans 797–953 to ^15^N-labeled hRpn13 to find binding [3]. As part of an attempt to further define the hRpn13-binding region on hRpn2, we generated a truncated peptide that spans 916–953, as described in Materials and Methods. We acquired 2D ^1^H, ^15^N NMR experiments on 0.6 mM free ^15^N labeled hRpn2 (916–953, Fig 1A, black) and with equimolar hRpn13 Pru domain (Fig 1A, orange). The NMR experiment revealed a subset of hRpn2 signals to shift significantly while others were unaffected by the presence of hRpn13 Pru (Fig 1A).

To determine which hRpn2 amino acids are involved in hRpn13 Pru binding, we used NMR experiments to assign each signal to an amino acid in hRpn2 (916–953) in its free state and hRpn13-complexed state, as described in Materials and Methods. We were able to assign all of the amide signals to hRpn2 amino acids, except for Q940 and E941. In addition, amino acids that remained at the N-terminal end from the PreScission protease tag (sequence: GPGS) were also assigned (Fig 1A, indicated with an asterisk). These assignments are depicted in Fig 1A, with the exception of E937 and E938, which were unambiguously assigned by using 3D NMR experiments, but are congested in the center of the 2D spectrum at ~8.45 and ~122 ppm in the ^1^H and ^15^N dimensions, respectively.

Comparison of the hRpn2 spectra with and without hRpn13 Pru domain illustrated large effects for F948, E949, Y950, and I951 (Fig 1A, indicated by solid arrows). We quantified the effect of hRpn13 addition across the hRpn2 sequence, as described in Materials and Methods, to find very little shifting for amino acids at the N-terminal end of hRpn2 (916–953) (Fig 1B). By contrast, significant shifting was calculated for E943-I951.

### An hRpn2-derived peptide binds to hRpn13 Pru with 12 nM affinity

**Fig 2:**
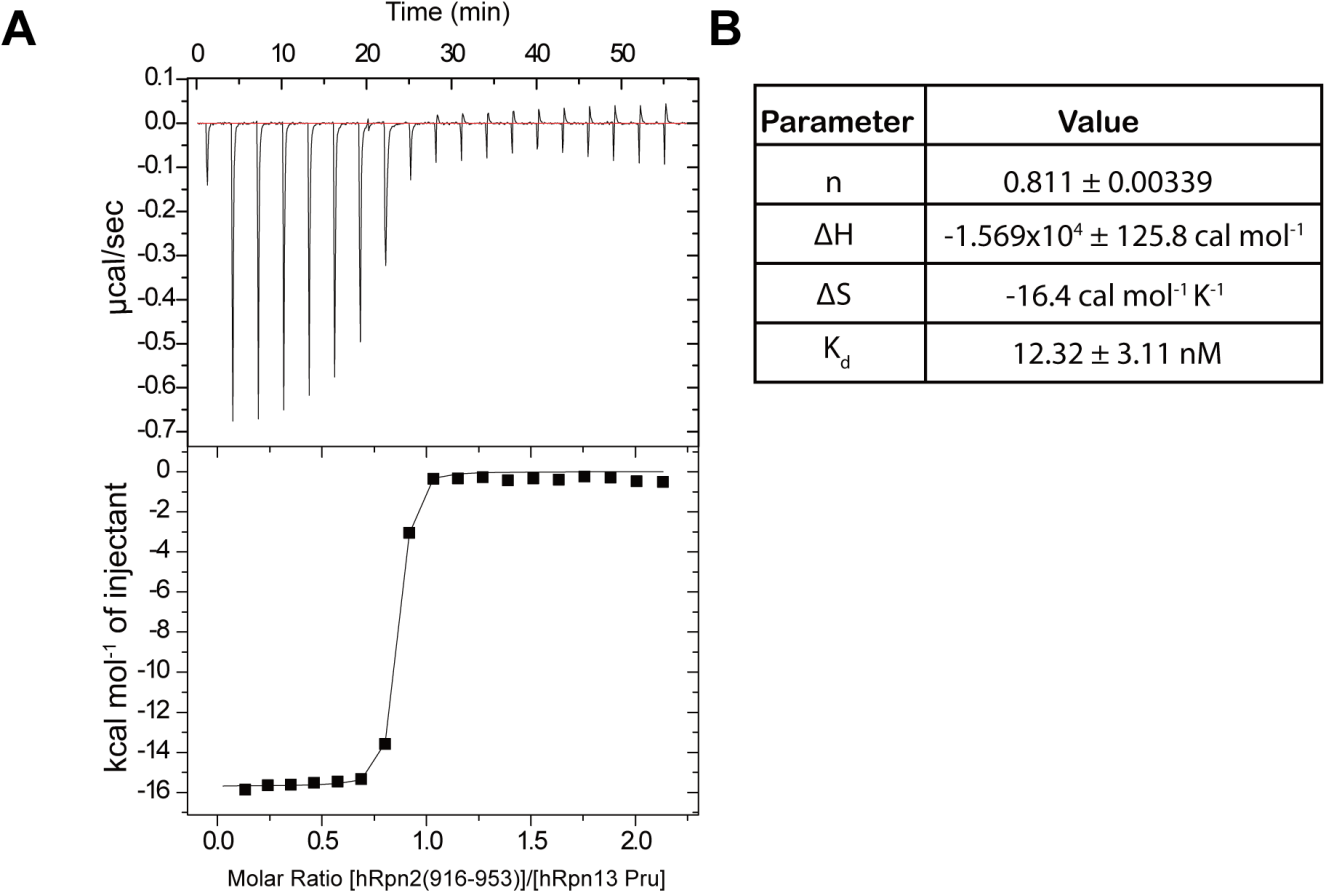
An hRpn2-derived peptide binds to hRpn13 Pru domain with 12 nM affinity. (A) 200 μM hRpn2 (916–953) was injected into a calorimeter cell that contained 20 μM hRpn13 Pru. The binding isotherm (top) was integrated to yield the change in enthalpy as a function of hRpn2 peptide (bottom). (B) The data fit well to a 1-site binding mode with the indicated thermodynamic values.

We next used isothermal titration calorimetry (ITC) to determine the affinity of hRpn13 Pru and hRpn2 (916–953). 200 μM hRpn2 (916–953) was injected into a calorimeter cell that contained 20 μM Rpn13 Pru domain. This initial injection was 0.5 μL, after which 2.1 μL was used. The thermogram indicated a high affinity binding profile with favorable enthalpy (Fig 2A). The data fit to a 1-site binding mode with a K_d_ value of 12 nM (Fig 2B). The mean concentration of cytosolic proteasome in intact neurons is estimated to be 190 nM [26], a concentration 15-fold greater than the K_d_ value for hRpn2 interaction with hRpn13.

### Structural properties of the hRpn13-binding region of hRpn2

**Fig 3:**
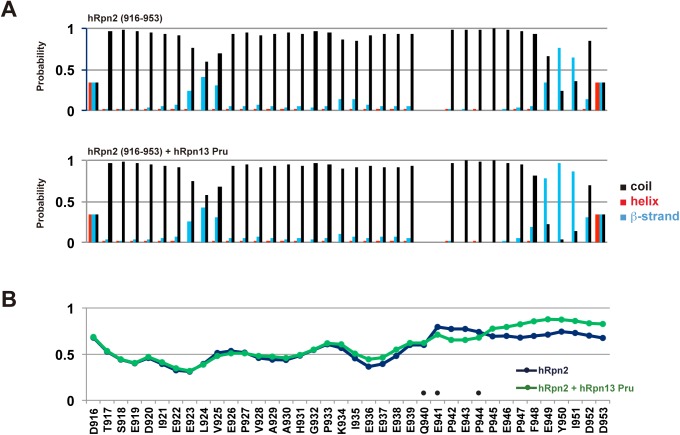
Structural characteristics of the hRpn2-derived peptide. (A) Probability of forming an α-helix (red), β-strand (blue) or coil (black), based on NMR signals for hRpn2 (916–953) (top) and its hRpn13-bound state (bottom). (B) Order parameters (S^2^) for hRpn2 (blue) and its Rpn13-bound state (green). In this analysis, a value below 0.5 is dynamic. This Fig and (A) were generated by TALOS+ by using NMR chemical shift assignments for N, HN, C’, Cα, and Cβ atoms. Q940, E941, and P944 were not assigned and are indicated with circles.

We used TALOS+ (http://spin.niddk.nih.gov/bax/software/TALOS) to predict the secondary structure and dynamic properties of hRpn2 in its free and Rpn13-bound state from our assigned N, NH, C’, Cα, and Cβ values. In both analyses, the peptide was predominately found to be unstructured (Fig 3A, black bars). However, amino acids involved in binding to hRpn13 Pru demonstrated a propensity to form a β-strand, especially when bound to hRpn13 (Fig 3A, blue bars). These include E949, Y950 and I951.

The region involved in binding to Rpn13 was also found to be less dynamic compared to the remainder of hRpn2. Most of the hRpn2-derived peptide was found to be dynamic, as indicated by S^2^ values of ~0.5 (Fig 3B). The Rpn13-binding region however showed higher S^2^ values, which increased in the presence of Rpn13 (Fig 3B).

From the dynamic region, two signals of unequal population were observed in the hRpn2 free state for A929 and I935, and in the hRpn13-bound state, V925, E926, and V928 also exhibited two sets of signals (Fig 1A). This phenomenon suggests that these five amino acids exchange between two distinct states. It is likely that this exchange is triggered by proline isomerization, as nearby P927 exhibited two sets of Cα, Cβ and C’ signals (data not shown). This proline was the only one in which we were able to observe two sets of signals.

### hRpn2 (916–953) is highly dynamic with E939-D953 becoming ordered upon binding to the hRpn13 Pru domain

**Fig 4:**
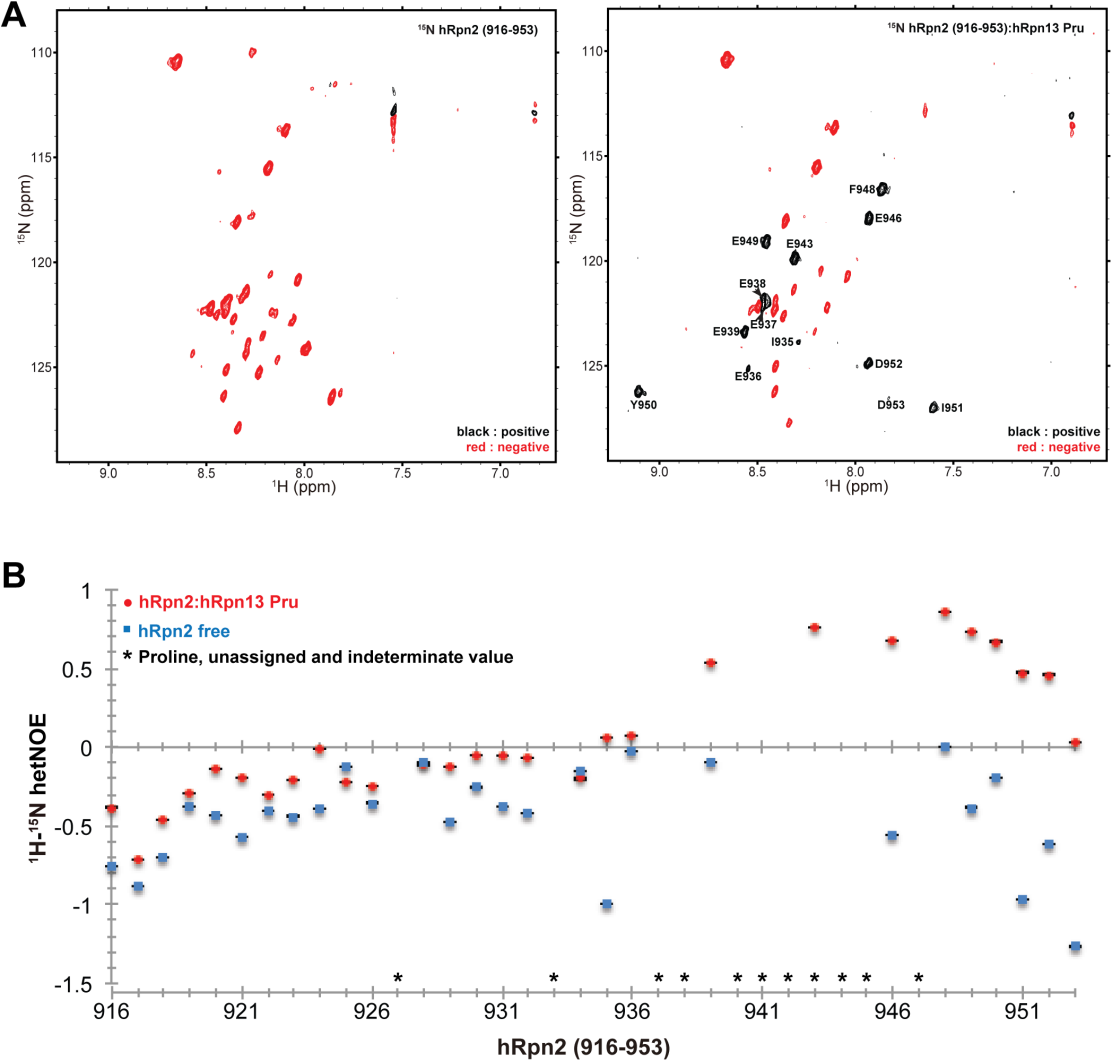
Highly dynamic hRpn2 (916–953) becomes ordered at the C-terminal region upon binding to hRpn13 Pru. (A) ^1^H-^15^N heteronuclear NOE spectra of hRpn2 (916–953) (left) and bound to hRpn13 Pru domain (right). Positive and negative signals are black and red, respectively. (B) Plots of heteronuclear NOE enhancement values (hetNOE) for hRpn2 (916–953) (blue squares) and hRpn2 (916–953):hRpn13 Pru (red circles). Prolines, unassigned residues (Q940 and E941), and those with too much overlap for reliable integration are indicated by asterisks. Error bars are small and indicated, as calculated by using relax.

To test further the dynamic properties of free and hRpn13-bound hRpn2 (916–953), we performed ^1^H, ^15^N heteronuclear NOE experiments with a four second saturation transfer or control period. The free protein fragment was found to be highly dynamic, with heteronuclear NOE enhancement values less than zero across the amino acid sequence (Fig 4A, left panel, and 4B, blue). Addition of hRpn13 Pru domain significantly increased these values for E939–D953 (Fig 4A, right panel, and 4B, red), indicating a loss of high frequency motions. The average heteronuclear NOE enhancement value for E939-D953 in the free and hRpn13-bound peptide was -0.51 and 0.58, respectively. By contrast, a smaller difference was observed in the region spanning 916–936, with an average heteronuclear NOE enhancement value of -0.43 and -0.19 in the free and bound states, respectively. Altogether, these data support the chemical shift perturbation study (Fig 1), and implicate the C-terminal region of hRpn2 spanning E939-D953 as the hRpn13 Pru domain-binding site.

### Strictly conserved F948 and neighboring Y950-I951 are required for hRpn2 peptide interaction with endogenous hRpn13 in 293T cells

**Fig 5:**
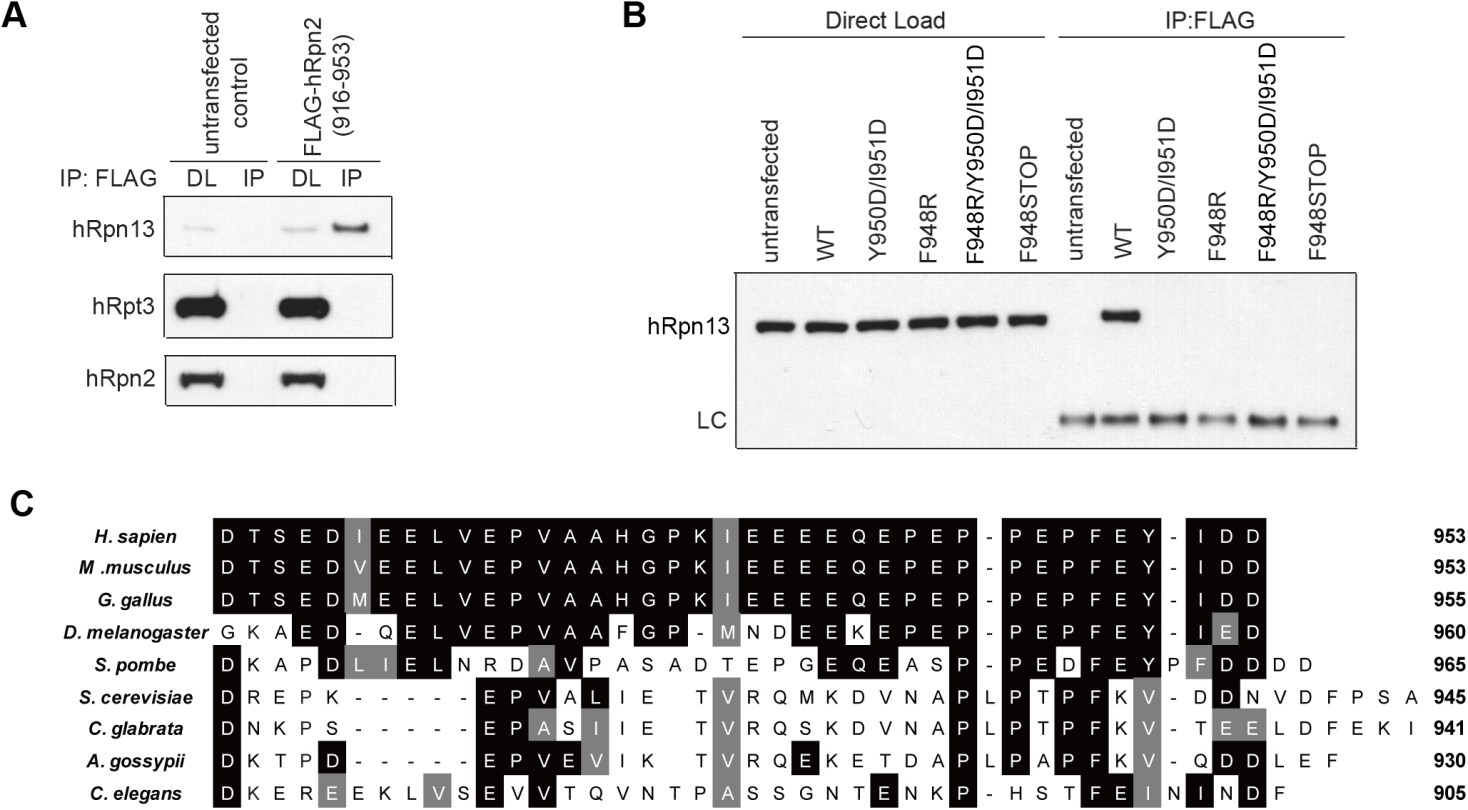
Strictly conserved F948 and Y950/I951 are required for hRpn2 interaction with hRpn13 in 293T cells. (A) 293T cells were transfected with or without 0.5 μg p3XFLAG-CMV7.1-hRpn2 (916–953) plasmid and the cell lysates immunoprecipitated by anti-FLAG antibody and immunoprobed for proteasome subunits hRpn13, hRpt3 and hRpn2, as indicated. (B) Cell lysates or immunoprecipitates from 293T cells expressing FLAG-hRpn2 (916–953) wild-type (WT) or with the indicated mutations were subjected to immunoprobing with anti-Rpn13 antibody, as indicated. LC, light chain; DL, direct load. An untransfected negative control is included. (C) Alignment across species for the C-terminal 38 amino acids of Rpn2. Strictly and moderately conserved amino acids are shaded in black and grey, respectively. This Fig was generated by using ClustalW2 (http://www.ebi.ac.uk/Tools/msa/clustalw2/).

To test whether hRpn2 (916–953) is competent for interaction with endogenous hRpn13 in a human cell line, we sub-cloned this fragment in frame with an N-terminal FLAG tag into the mammalian expression vector p3XFLAG-CMV7.1. The resulting plasmid was transfected into 293T cells and the cell lysate immunoprecipitated with anti-FLAG antibody. The immunoprecipitate was immunoprobed for hRpn13 by using anti-Rpn13 antibody, which confirmed the interaction of the hRpn2-derived peptide with endogenous hRpn13 in 293T cells (Fig 5A, top panel).

Based on our NMR titration experiment (Fig 1), we hypothesized that hRpn2 F948, Y950, and I951 are important for hRpn13 interaction. We thus replaced F948 with arginine or a stop codon and separately, replaced Y950 and I951 with aspartic acid. These FLAG-hRpn2 variants were transfected into 293T cells along with FLAG-hRpn2 (916–953) and subjected to immunoprecipitation with anti-FLAG antibody followed by immunoprobing with anti-Rpn13 antibody, as described for Fig 5A. All three of these substitutions resulted in a peptide that was unable to immunoprecipitate endogenous hRpn13 from 293T cells (Fig 5B). The importance of F948 and Y950 is further supported by their conservation among eukaryotic Rpn2 species; F948 is strictly conserved whereas Y950 is highly conserved (Fig 5C).

### hRpn2 (916–953) displaces hRpn13 from proteasome in 293T cells

We rationalized that if the 916–953 region in hRpn2 is used to dock hRpn13 into the proteasome, then hRpn13 would not be able to interact with the peptide while seated in the proteasome. We thus tested whether the hRpn2-derived peptide can immunoprecipitate endogenous proteasome subunits hRpn2 and hRpt3 after immunoprecipitation with anti-FLAG antibody. This experiment demonstrated neither of these RP components was immunoprecipitated by the peptide (Fig 5A, bottom two panels). Thus, hRpn13 immunoprecipitated by the hRpn2-derived peptide is not bound to the proteasome.

**Fig 6:**
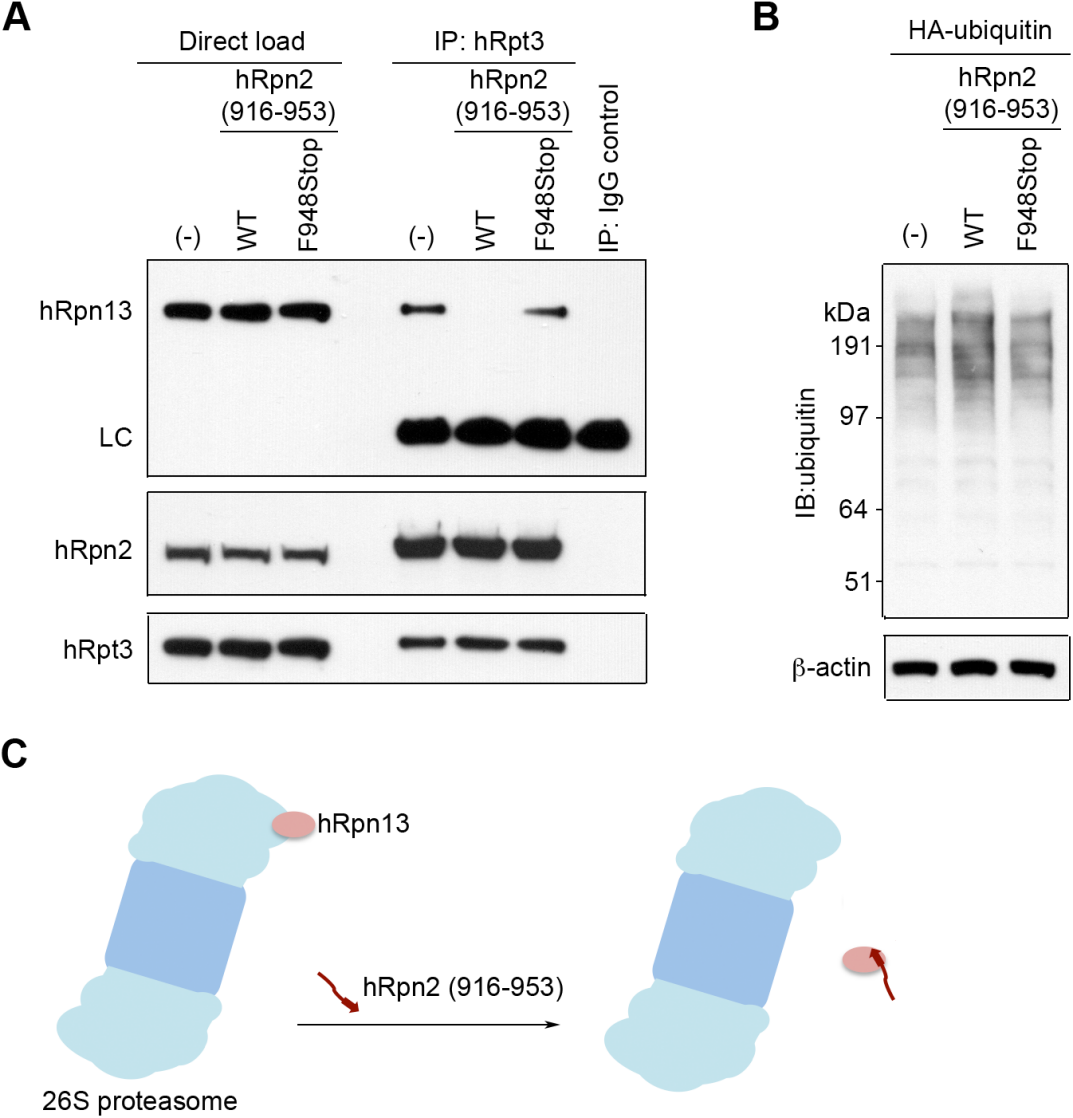
The hRpn2-derived peptide depletes hRpn13 from the proteasome. (A) 293T cells were transfected with p3XFLAG-CMV7.1-hRpn2 (916–953) WT or F948Stop plasmids and the cell lysates immunoprecipitated with anti-hRpt3 antibodies and immunoprobed with antibodies against hRpn13, hRpt3, and hRpn2, as indicated. (B) Cell lysates from 293T cells transfected with HA-ubiquitin alone or together with either p3XFLAG-CMV7.1-hRpn2 (916–953) WT or F948Stop plasmids were immunoblotted with anti-ubiquitin and anti-β-actin antibodies, as indicated. (C) A model illustrating hRpn2 (916–953) peptide interacting with hRpn13 and displacing it from the proteasome.

We next tested whether over-expression of the hRpn2-derived peptide depletes hRpn13 from the proteasome. For this purpose, we immunoprecipitated proteasome by anti-hRpt3 antibody and detected the presence of hRpn13 by immunoprobing with anti-hRpn13 antibody. Whereas hRpn13 co-immunoprecipitated with anti-hRpt3 antibody in untransfected 293T cells, expression of hRpn2 (916–953) peptide significantly reduced the amount of hRpn13 at the proteasome (Fig 6A, top panel, compare lane 4 with lane 5). Expression of the F948Stop mutant of this peptide, which does not bind hRpn13 (Fig 5B), does not deplete hRpn13 from proteasome (Fig 6A, lane 6). As expected, the assembly of hRpn2 into the proteasome was not affected by expression of the hRpn2-derived peptide (Fig 6A, second panel).

We next tested whether over-expression of the hRpn2-derived peptide in 293T cells leads to the accumulation of ubiquitinated proteins. Lysates from cells transfected with HA-ubiquitin alone or together with either FLAG-hRpn2 (916–953) WT or F948Stop were subjected to immunoblotting with anti-ubiquitin antibody. Cells expressing F948Stop were used as a negative control, and immunoblotting with anti-actin antibody to confirm equivalent loading. This experiment revealed an increase in the presence of ubiquitinated proteins in the cell lysate for 293T cells over-expressing the hRpn2 WT-derived peptide (Fig 6B). This increase was not observed for the negative control peptide (F948Stop) that does not bind to Rpn13.

## Discussion and Conclusions

Here, we demonstrate that a 38-amino acid peptide derived from the C-terminal end of hRpn2 binds to hRpn13 with high affinity (Fig 2). We identify the key interacting amino acids by using NMR spectroscopy and find this region to be less dynamic than the remainder of the peptide, and to have a propensity toward forming a β-strand (Fig 3). Importantly, we find that this peptide is capable of binding to full-length hRpn13 in mammalian cells and that replacement of a strictly conserved phenylalanine with arginine abrogates this interaction (Fig 5). This phenylalanine is not sufficient for binding however, as aspartic acid substitution of Y950 and I951 also loses hRpn2 peptide interaction with hRpn13.

We find that the hRpn2-derived peptide displaces hRpn13 from the proteasome (Fig 6). This finding highlights the importance of Rpn2 in docking Rpn13 to the proteasome. It also demonstrates the utility of this peptide as a tool for specifically studying the function of hRpn13 at the proteasome.

The proteasome is a therapeutic target with inhibitors that act on the catalytic CP approved for certain cancer types. Recent efforts have focused on strategies that target the 19S RP and Rpn13 appears to be a putative target [18]. The peptide reported here binds tightly to hRpn13 and displaces it from the proteasome; thus, it may serve as a potential lead for a new class of hRpn13 inhibitors.
